# Effect of Formaldehyde on Asthmatic Response to Inhaled Allergen Challenge

**DOI:** 10.1289/ehp.9414

**Published:** 2006-11-07

**Authors:** Véronique Ezratty, Marcel Bonay, Catherine Neukirch, Gaëlle Orset-Guillossou, Monique Dehoux, Serge Koscielny, Pierre-André Cabanes, Jacques Lambrozo, Michel Aubier

**Affiliations:** 1 Service des Etudes Médicales d’EDF et de Gaz de France, Paris, France; 2 Unité 700, INSERM, Faculté Xavier Bichat, Paris, France; 3 Service de Physiologie-Explorations Fonctionnelles; 4 Service de Pneumologie; 5 Clinical Centre of Investigation INSERM 007 and; 6 Biochimie A, Hôpital Bichat-Claude Bernard, Paris, France; 7 Department of Biostatistics, Institut Gustave Roussy, Villejuif, France

**Keywords:** allergen, asthma, formaldehyde, human exposure study

## Abstract

**Background:**

Exposure to formaldehyde may lead to exacerbation of asthma.

**Objectives:**

Our aim in this study was to investigate whether exposure to a low level (500 μg/m^3^) of formaldehyde enhances inhaled allergen responses.

**Methods:**

Twelve subjects with intermittent asthma and allergy to pollen were exposed, at rest, in a double-blind crossover study to either formaldehyde or purified air for 60 min. The order of exposure to formaldehyde and air-only was randomized, and exposures were separated by 2 weeks. We also performed an allergen inhalation challenge after each exposure. Airway responsiveness to methacholine and lower airway inflammation (induced sputum) were assessed 8 hr after allergen challenge.

**Results:**

The median dose of allergen producing a 15% decrease in forced expiratory volume in 1 sec (PD_15_FEV_1_) was 0.80 IR (index of reactivity) after formaldehyde exposure compared with 0.25 IR after air-only exposure (*p* = 0.06). Formaldehyde exposure did not affect allergen-induced increase in responsiveness to methacholine (*p* = 0.42). We found no formaldehyde-associated effect on the airway inflammatory response, in particular the eosinophilic inflammatory response, induced by the allergen challenge 8 hr before.

**Conclusion:**

In this study, exposure to 500 μg/m^3^ formaldehyde had no significant deleterious effect on airway allergen responsiveness of patients with intermittent asthma; we found a trend toward a protective effect.

Formaldehyde is a well-known airborne contaminant causing eye, nose, and throat irritation as well as airway irritation and slight neuropsychologic changes (Hester and Harrison 1998; Samet et al. 1988).

The major indoor sources of formaldehyde are off-gassing from urea–formaldehyde foam insulation, particle board, paneling, plywood, some carpets and furniture, and, to a lesser extent, tobacco smoke and indoor combustion sources. Indoor concentrations of formaldehyde can vary between different countries (Sakaï et al. 2004). In a Japanese study, formaldehyde concentrations ranged between 91.25 and 290 μg/m^3^ (Minami et al. 2002), whereas in the United Kingdom, the highest level measured in 876 homes was much lower (median = 24 μg/m^3^) (Brown et al. 2002).

Indoor formaldehyde concentrations measured in mobile homes in the United States ranged from nondetectable values to 575 μg/m^3^ (Liu et al. 1991). Indoor concentrations generally exceed those outdoors, and studies on formaldehyde levels in homes have demonstrated higher formaldehyde concentrations in newer compared with older dwellings, with higher levels in buildings built after 1970 (Gilbert et al. 2005).

Formaldehyde is an etiologic factor in occupational asthma. However, although formaldehyde may cause asthma in some individuals, this occurs relatively rarely (Nordman et al. 1985; Paustenbach et al. 1997).

Whether nonoccupational exposure to formaldehyde is related to asthma is still subject to discussion (Delfino 2002; Institute of Medicine 2000). In murine models, formaldehyde exposure has been shown to enhance the allergic eosinophilic airway inflammation in sensitized mice (Sadakane et al. 2002). In a part of the European Community Respiratory Health Survey, asthma prevalence was greater for newly painted homes, consistent with greater differences in formaldehyde exposure (Wieslander et al. 1997). A relationship between physician-diagnosed asthma and indoor concentration of formaldehyde was reported even at low levels of exposure in children (Rumchev et al. 2002). Franklin et al. (2000) reported that exposure to formaldehyde in homes could produce a subclinical inflammatory response in the airways of healthy children. A possible association between exposure to formaldehyde and allergic sensitization to common aeroallergens has been suggested by another cross-sectional study in children (Garrett et al. 1999).

Human exposure studies can provide valuable data for assessing more specifically the acute effects of air pollutants, particularly the airway response to allergen (Sandström 1995). The hypothesis that formaldehyde enhances asthmatic response to allergen has not yet been investigated in controlled conditions in humans. To test this hypothesis, we carried out this controlled human study to investigate the effect of a short exposure to 500 μg/m^3^ formaldehyde on asthmatic response to inhaled allergen.

## Methods

### Subjects

Twelve subjects (seven men and five women) participated in the study (Table 1). All of the subjects were between 18 and 44 years of age (median = 25 years) and had been diagnosed with intermittent asthma and allergy to pollen.

The diagnosis of intermittent asthma was based on reversible attacks of dyspnea less than twice per week and attacks of night respiratory problems, with a peak expiratory flow (PEF) > 80% of predicted value and/or normal pulmonary function test, less than twice per month. All subjects were allergic to grass pollen, as determined by history of seasonal asthma symptoms and allergy skin testing. All subjects used inhaled β_2_-agonist as needed, and nine used antihistamine (anti-H_1_) medications during the pollen season. None were receiving anti-inflammatory therapy or other current treatments. The study was performed outside the grass pollen season. All subjects were nonsmokers.

Before the the exposure experiments began, each subject underwent a physical examination. Also, seasonal allergy to grass pollen was confirmed by positive skin prick test performed using a standardized extract including five grass pollen allergens: *Dactylis glomerata, Anthoxanthum odoratum, Lolium perenne, Poa pratensis,* and *Phleum pratense* (Phl p5) (Stallergenes Laboratory, Antony, France). Skin prick test responses for allergens were considered positive if the wheal diameter was at least 3 mm greater than that for the negative control and at least 50% of the diameter of the positive control. Blood samples were obtained for analysis of total IgE and eosinophils in serum. Pulmonary function tests were performed and sputum was collected. All subjects were free from upper respiratory infections for at least 4 weeks before the study. Before enrollment in the study, all participants gave written informed consent. The study was approved by the ethical committee of Saint-Germainen-Laye-Hospital (project 00019, registered on 9 May 2000).

### Study protocol

In a crossover design study, each subject was exposed at rest to filtered air or to a concentration of 500 μg/m^3^ (0.4 ppm) formaldehyde for 60 min on two separate days. The exposures were performed at the same hour (0700 hours) and occurred on the same day of the week, with an interval of 2 weeks between exposure. The order of exposure to formaldehyde and air-only was double-blinded and randomized. The only member of the research team aware of the type of exposure was the engineer in charge of the injection of formaldehyde into the chamber. The nature of exposure was made known to the other members of the team only after completion of the statistical analysis.

Lung function was measured with a spirometer according to the European Community Respiratory Health Survey specifications; measurements were taken immediately before, during, and 8 hr after the end of the allergen challenge. Forced expiratory volume in 1 sec (FEV_1_) and PEF were measured with a portable combined spirometer every 15 min during the exposure to formaldehyde or air-only in the chamber and every hour until the methacholine provocation test, which was performed 8 hr after the end of the allergen bronchial challenge.

### Formaldehyde/clean air exposure

A 8.8-m^3^ exposure chamber was installed at the Hospital Bichat in Paris. The chamber was supplied with fresh, particle-free air at a mean temperature of 25°C and a mean relative humidity of 32%. The air supply passed through both HEPA and activated carbon filters. The formaldehyde atmosphere was created by injecting and diluting saturated vapors from a heated solution of formaldehyde at the exit of the filtration box; these vapors flowed into the ventilation diffuser located in the center of the chamber ceiling. A continuous 1-hr injection of the formaldehyde solution was sufficient to reach a steady state. The formaldehyde concentration in the chamber was monitored continuously with semiconductor gas sensor technology during the experiments to ensure that there was no fluctuation in formaldehyde levels during exposure. The air ejected from the chamber was evacuated outside the building without recirculation.

### Allergen bronchial challenge

Each exposure to formaldehyde or air-only was immediately followed by an allergen inhalation challenge. This challenge involved an automatic inhalation-synchronized Mefar MB3 dosimeter jet nebulizer (Mefar SpA, Bovezzo, Italy). We used the same standardized extract of five grass pollen allergens as for the skin test (Stallergenes Laboratory) The initial allergen concentration of standardized pollen extract was 0.1 or 0.2 IR (index of reactivity), as previously described by Aubier et al. (1998). The concentration of inhaled allergen was doubled every 15 min; the FEV_1_ was measured immediately after each doubling and again 10 min after each inhalation. The dose of allergen producing a 15% decrease in the FEV_1_ was defined as the PD_15_FEV_1_. If the FEV_1_ had fallen by ≥ 10%, we required that it be measured again every 5 min until no further decrease was observed. Once it reached that point, inhalation of a higher concentration could continue. No further allergen was given *a*) when FEV_1_ had fallen by ≥ 15%; *b*) when the highest dose of 2 IR was reached (in that case PD_15_FEV_1_ was considered equal to 2 IR); or *c*) if respiratory symptoms occurred. Graphical representations of FEV_1_ and PEF according to time were performed during the 8 hr following allergen bronchial challenge for each of the 24 exposures. PD_15_FEV_1_ was estimated without knowing which arm was the treatment arm.

### Pulmonary function and methacholine-challenge testing

We measured responsiveness to methacholine 8 hr after the allergen bronchial challenge ended. All tests were performed with the same dosimeter used for allergen inhalation. The nebulizers were changed after each test. Flow-volume curves were obtained with a Biomedin spirometer (Biomedin Srl, Padova, Italy) in order to determine FEV_1_, forced vital capacity (FVC), forced expiratory flow between 25% and 75% of the vital capacity, and PEF. The spirometry technique met international standards, and references values were those of the European Respiratory Society (Quanjer et al. 1993). Results are given as percentages of predicted values. We assessed airway responsiveness by methacholine challenge testing using an automatic inhalation-synchronized Mefar MB3 dosimeter jet nebulizer (Mefar SpA., Bovezzo, Italy) as previously described by Aubier et al. (1992). After inhalation of isotonic saline as a control, subjects were administered methacholine until the FEV_1_ had dropped by ≥ 20% from the post-saline value, or until the maximum cumulative dose of 4 mg had been given. The cumulative doses administered were 0.0156, 0.0625, 0.25, 1.0, 2.0, and 4.0 mg. A 3-min interval was allowed before each dose increment. FEV_1_ was measured 1 min after each dose; we used the best of three acceptable measurements to create dose–response curves. The methacholine provocative dose (PD) causing a 20% decrease in FEV_1_ from control FEV_1_ (PD_20_ methacholine) was determined by interpolation from the dose–esponse curve (Chai et al. 1975).

### Sputum induction and measurement of inflammatory markers

Sputum induction was performed at baseline and immediately after the methacholine challenge with an aerosol of hypertonic saline, following the method of Pin et al. (1992). At the beginning of the test and before each period of inhalation, FEV_1_ was measured for safety. The aerosol was generated by a Syst’am ultrasonic nebulizer (System Assistance Medical, Villeneuve sur Lot, France) with increasing concentrations of saline (3, 4, and 5%) inhaled via a mouthpiece for 5-min periods for up to 30 min. Patients were then asked to rinse their mouth, blow their nose, and cough sputum into a sterile container.

The sputum was examined within 1 hr using a modified method described by Pizzichini et al. (1996). The entire sputum sample was poured into a Petri dish and inspected for salivary contamination under an inverted microscope; all portions that appeared free of salivary contamination were placed in a preweighed 15 mL polystyrene tube using forceps. Dithiothreitol (0.1%; Sigma, St. Quentin Fallavier, France) was freshly diluted in distilled water equal to 4 times the sputum weight and added to the sputum sample. The mixture was vortexed for 30 sec and placed on a bench rocker and rocked for 15 min. A further 4 volumes of Dulbecco’s phosphate-buffered saline was added to stop the effect of dithiothreitol and rocked for 5 min. The suspension was filtered through a 70-μm cell strainer. The resulting suspension was centrifuged at 800 × *g* for 10 min, and the supernatant was aspired and stored in Eppendorf tubes at –70°C in the presence of aprotinin.

Total nonsquamous cell counts were performed in a hemocytometer and expressed as millions per milligram of selected induced sputum. The proportion of salivary squamous cells was noted, and cell viability was determined using the trypan blue exclusion method. From the remainder of the filtrate, 10 cytospins were prepared, air-dried, and fixed. Differential cell counts were performed by counting 400 cells on May Grünwald Giemsa–stained slides. Results were expressed as a percentage of the total nonsquamous count. Slides were coded, and cell counts were performed by an expert observer who did not know the clinical characteristics of the patients. Only samples with cell viability > 70% and squamous cell contamination < 20% were considered adequate.

We measured sputum supernatant concentrations of interleukins (IL-1, IL-4, IL-5, IL-8, IL-10), granulocyte–macrophage colony-stimulating factor (GM-CSF), monocyte chemotactic protein-1 (MCP-1), tumor necrosis factor- α (TNF- α ), interferon-γ (IFN-γ ), and eotaxin-1 using commercially available ELISAs (R&D Systems, Abingdon, UK) according to the manufacturer’s instructions. The lower detection limits of the assays were as follows: IL-1, 0.1 pg/mL; IL-4, 0.13 pg/mL; IL-5, 3 pg/mL; IL-8, 10 pg/mL; IL-10, 0.5 pg/mL; GM-CSF, 0.25 pg/mL; MCP-1, 5 pg/mL; TNF- α , 0.12 pg /mL; IFN-γ , 8 pg/mL; and eotaxin-1, 5 pg/mL.

Eosinophil cationic protein levels (ECP) were measured by a commercially available enzyme assay (CAP-FEIA, Pharmacia, St. Quentinen-Yvelines, France), with a lower detection limit of 2 ng/mL.

### Questionnaire and postexposure follow-up

After 0, 15, 30, 45, and 60 min of exposure to formaldehyde or air-only in the chamber, the subjects were asked 14 questions concerning respiratory symptoms and perception of discomfort (i.e., perception of an odor, eye irritation, nose/throat irritation, chest discomfort/tightness, coughing, shortness of breath, nausea, dyspnea, headache, fatigue, dizziness, other discomfort).

Subjective symptoms and medication were also recorded. Each subject measured FEV_1_ and PEF twice daily with a portable combined spirometer during the 2-week interval after each exposure.

### Statistical analysis

We analyzed differences between two exposures (either between exposures with formaldehyde and air, or between the first exposure and the second exposure after the washout) using the Wilcoxon’s non-parametric sign rank test. *p*-Values < 0.05 were considered significant.

## Results

All 12 subjects completed the two exposures and all of the allergen and methacholine challenges. Four subjects reported minor complaints during exposure to both air-only and formaldehyde. One reported nose irritation during air-only exposure, and another subject reported having a runny nose during formaldehyde exposure but no symptoms or discomfort during air exposure. No distinct odor was reported by any subject during exposure to air-only or formaldehyde. No major clinical adverse reaction was observed.

Exposure of allergic asthmatic patients at rest to 500 μg/m^3^ formaldehyde for 1 hr had no direct effect on respiratory function either during or immediately after the exposure session. The FVC and FEV_1_ values, measured immediately after formaldehyde exposure, were not significantly different from those obtained after air-only exposure.

### Allergen bronchial challenge

**Figure 1:**
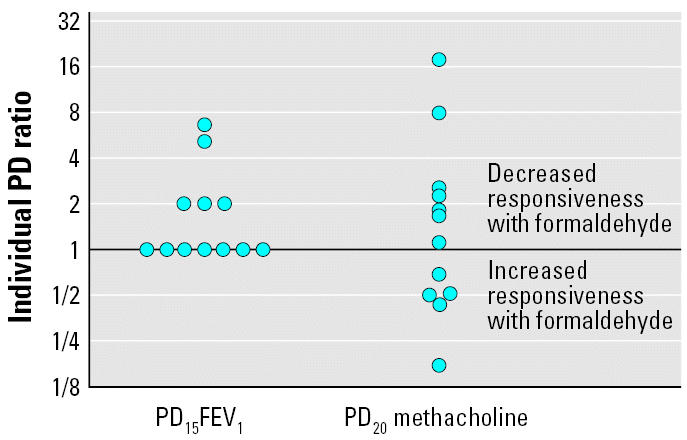
Ratios between PD15FEV_1_ and PD_20_ methacholine measurements for each subject (after formaldehyde exposure divided by after air-only exposure). The ratios between the PD_15_FEV_1_ measurements are always ≥ 1, showing an unchanged or a decreased allergen responsiveness with formaldehyde compared with air-only. The ratios between the PD_20_ methacholine measurements after exposure to formaldehyde and air-only range from 0.15 to 16.

Airway responsiveness to allergen was measured using the PD_15_FEV_1_. Formaldehyde versus air-only exposure resulted in a PD_15_FEV_1_ that was higher in five patients and unchanged in seven (Figure 1). The median PD_15_FEV_1_ was 0.80 (range, 0.15–2.0) IR after formaldehyde exposure compared with 0.25 (range, 0.1–2.0) IR after air-only exposure (*p* = 0.06) (Table 2). We observed no “order effect” concerning PD_15_FEV_1_: results were not significantly different between the first exposure to formaldehyde or air-only (no wash-out) and the second exposure (after a wash-out).

### Methacholine bronchial challenge

Methacholine responsiveness was assessed 8 hr after the end of the allergen challenge. Formaldehyde versus air-only exposure resulted in a PD_20_ methacholine that was lower in three subjects, higher in four, and unchanged (within a doubling dose) in five (Figure 1). Formaldehyde exposure did not affect the allergen-induced increase in responsiveness to methacholine (median PD_20_, 0.17 mg after formaldehyde vs. 0.23 mg after air-only exposure; *p* = 0.42) (Table 2). No “order effect” was observed.

### Sputum sample analysis

Eosinophils, ECP, and MCP-1 increased significantly in induced sputum 8 hr after the allergen challenge compared with levels measured at baseline.

The percentage of neutrophils and eosinophils in induced sputum obtained after formaldehyde exposure was not statistically different from that obtained after air-only exposure. The level of all the parameters measured in sputum supernatant obtained after formaldehyde exposure was not significantly different from that obtained after air-only exposure (Table 3).

During the 2 weeks after each exposure, subjective symptoms and peak flow measurements did not differ significantly between subjects who were exposed to air-only and those who were exposed to formaldehyde.

## Discussion

Several epidemiologic studies (Franklin et al. 2000; Garrett et al. 1999; Rumchev et al. 2002; Wieslander et al. 1997) have suggested possible associations between formaldehyde exposure and either asthma or allergic sensitization to common aero allergens. These cross-sectional studies assessed chronic exposure to low levels of formaldehyde. Concerning the effect of acute exposures to formaldehyde on allergic response, the only data available were reported in a murine model (Sadakane et al. 2002). Several studies have been performed with air pollutants to assess interaction with allergenic response; some have shown that asthmatic response could be enhanced by a brief preexposure to air pollutants, in particular, nitrogen dioxide or ozone (Barck et al. 2005; Jörres et al. 1996; Molfino et al. 1991; Strand et al. 1997; Tunnicliffe et al. 1994).

The hypothesis that a brief exposure to ambient levels of formaldehyde enhances asthmatic response to allergen has not yet been reported in controlled human exposure studies. The aim of this study was to examine whether a 1-hr exposure to 500 μg/m^3^ formaldehyde enhances the asthmatic response to inhaled pollen allergen in subjects with intermittent asthma. We chose this level of formaldehyde to remain within realistic conditions while maximizing our chances to demonstrate an adverse effect. Mean indoor formaldehyde concentrations are usually < 500 μg/m^3^, although such a concentration can be found in indoor environments (Institute for Environment and Health 1996).

Formaldehyde exposure alone did not cause any change in lung function, which is in accordance with earlier reports that concluded that lung function of healthy nonsmokers and asthmatics was generally unaffected by exposure to formaldehyde at levels ≤ 3,700 μg/m^3^ (Sauder et al. 1987).

We found no significant differences between the bronchial allergen responses after formaldehyde exposure compared with exposure to air-only. However, there was a tendency toward a lower immediate bronchial allergen response after exposure to formaldehyde compared with air-only, contrary to expectations. This result is not compatible with an adverse effect of formaldehyde on asthmatic response in the conditions tested and might suggest a protective effect. Such an effect was reported in mice preexposed to low concentrations of nitrogen dioxide (Hubbard et al. 2002; Proust et al. 2002). Moreover, Fujimaki et al. (2004) showed a decreased production of IL-1β in ovalbumin in immunized mice after exposure to a low dose of formaldehyde.

We assessed the effect of formaldehyde using conditions that minimize the possibility of bias: the order of exposures to formaldehyde or purified air was both randomized and double blinded. Subjects were tested in the same controlled conditions and with a constant level of air pollutants, temperature, and humidity. The delay between exposures was consistent with the literature concerning this type of study (Strand et al. 1997). The longer the wash-out period, the higher the risk of developing respiratory infections; we considered 2 weeks a good compromise between the risk of bias because of a late reaction after the allergen challenge and the risk of exclusion because of infection. Furthermore, if the delay between exposures had had an effect, we would have found different results between the first exposure to formaldehyde or air-only (no wash-out) and the second exposure to formaldehyde or air, which was not the case (i.e., no “order effect”).

Post hoc calculations showed that the power of the study was sufficient (> 80%) to show a significant difference if there was a 2-fold variation in PD_15_FEV_1_ between the two arms. We observed an increase in PD_15_FEV_1_ after formaldehyde exposure compared with air-only exposure (Figure 1). The increase was near statistical significance (two-sided, *p* = 0.06). The true value of the variation in PD_15_FEV_1_ may correspond to a decreased responsiveness with formaldehyde compared with air-only or to no change. However, in spite of the low number of patients, the power of the study is sufficient to conclude that the probability for an increased responsiveness with formaldehyde is very low (3%). Moreover, if there was an increased responsiveness, the increase would probably be so small that it would be impossible to demonstrate, even with a very large study.

Corren (1992) showed that a late bronchial response occurs 2 to > 12 hr after allergen exposure. In the present study, methacholine challenge and induced sputum tests were performed 8 hr after the end of allergen bronchial challenge, approximately when the maximum airway inflammatory reaction to allergen occurs. We observed no significant modification in airway responsiveness to methacholine after formaldehyde exposure at this time (8 hr after exposure). To assess airway inflammation, bronchial biopsy remains the gold standard. However, this process is invasive compared with induced sputum, which has proven to be a reproducible, sensitive, and valid method for the assessment of airway inflammation (Wilson 2002). Induced sputum has been used to detect cytokines in patients with bronchial asthma, and the up-regulation of cytokines in the airways can be assessed using noninvasive techniques, including sputum induction (Taha et al. 2001). In the present study, we measured in induced sputum several inflammatory cytokines and mediators that are well-known to be involved in the physiopathology of asthma. Formaldehyde exposure did not significantly affect inflammatory cytokines and mediators measured in sputum 8 hr after the end of the bronchial allergen challenge. However, the total dose of allergen required to reach the expected respiratory effect was higher after formaldehyde exposure than after air-only exposure (0.8 IR vs. 0.25 IR). A potential effect of formaldehyde on the response to methacholine challenge could have been masked because of the differences in allergen exposure between the two arms. It also applies for the airway inflammatory response.

Our study included patients with intermittent asthma who were not taking any anti-inflammatory therapy; although we observed no effect in this particular group of patients, this does not necessarily mean that the results can be generalized to patients with more severe asthma. Therefore, additional research is needed to examine effects among individuals with severe asthma.

To our knowledge, this is the first controlled human study examining possible interactions between formaldehyde exposure and allergen on asthmatic response. In this study, exposure to 500 μg/m^3^ formaldehyde did not enhance the asthmatic response to allergen. We even observed a trend to a protective effect. Future studies assessing effects of formaldehyde at higher doses, or with repeated or longer exposures, are needed to clarify interactions between formaldehyde and allergens in airways of patients with asthma.

## Figures and Tables

**Table 1:** Characteristics of subjects.

Subject	Age (years)	Sex	Asthma duration (year)	Smoking	FEV_1_ at inclusion (% pred)
1	34	M	18	N	100
2	33	F	19	N	101
3	45	M	10	N	109
4	18	M	12	N	105
5	24	M	8	N	103
6	28	F	10	E	111
7	26	F	20	N	95
8	37	F	15	N	109
9	25	M	20	N	101
10	26	F	18	N	93
11	21	M	6	N	108
12	26	M	14	N	89

Abbreviations: E, exsmoker; F, female; M, male; N, never smoker; % pred, percent predicted.

**Table 2:** Results [median (range)] of allergen bronchial challenge performed immediately after exposure to formaldehyde or air-only and methacholine bronchial challenge performed 8 hr after exposure.

	Exposure		
	Formaldehyde	Air-only	*p*-Value
Allergen challenge (PD_15_FEV_1_)	0.80 (0.15–2.0)	0.25 (0.10–2.0)	0.06
Methacholine challenge (PD_20_)	0.23 (0.01–3.6)	0.17 (0.03–4)	0.42

*p*-Values were determined by signed rank test.

**Table 3:** Results [median (range)] for parameters measured in sputum.

		Exposure		
	Baseline	Formaldehyde	Air-only	*p*-Value^a^
Total no. of cells	244 (213–496)	255 (215–633)	258 (229–438)	0.50
Bronchial cells (%)	14.4 (1.7–46)	4.4 (0.30–40)	3.5 (0.20–33)	0.82
Macrophages (%)	27 (3–57)	27.4 (2.8–79)	17.3 (2–82)	0.57
Lymphocytes (%)	0.3 (0–2.2)	1 (0–7)	0.4 (0–1.7)	0.31
Neutrophils (%)	58 (3.3–94)	32 (0–81)	34 (3–92)	0.73
Eosinophils (%)^b^	2.1 (0–31)	11.3 (0.8–89)	13.2 (3–81)	0.91
ECP (ng/mL)^b^	57 (3.8–130)	130 (3.9–200)	105.5 (41–200)	0.92
Eotaxin (pg/mL)	0 (0–0)	0 (0–14)	0 (0–15)	1.00
GM-CSF (pg/mL)	0 (0–1.6)	0 (0–0.69)	0 (0–7.87)	0.12
IFN-γ (pg/mL)	0 (0–23)	0 (0–14)	4 (0–14)	0.58
IL-1 (pg/mL)	10.5 (1.9–30)	11.5 (6–30)	7.5 (3–30)	0.90
IL-4 (pg/mL)	0.19 (0–2.5)	0.17 (0–0.85)	0.06 (0–1.7)	0.74
IL-5 (pg/mL)	0 (0–13)	4.5 (0–18)	4 (0–16)	0.82
IL-8 (pg/mL)	494 (17–1,312)	675 (69–1,200)	714.5 (81–2,500)	0.47
IL-10 (pg/mL)	1.7 (0–5.5)	1.4 (0–8.6)	3.45 (0–8.9)	0.75
MCP-1 (pg/mL)^b^	11 (0–72)	29 (0–108)	26.5 (0–129)	0.52
TNF- α (pg/mL)	0.26 (0–3.4)	0.16 (0–1.3)	0.26 (0–3.6)	0.20

a *p*-Values were determined by signed rank test and indicate comparison of formaldehyde to air-only.

b Significant increase between baseline and 8 hr after the end of the allergen challenge, whether the subject was exposed to air-only or to formaldehyde (*p* < 0.05).
